# Differential Effects of Antibiotics on the Horizontal Transfer of *bla*KPC Plasmid in Clinical *Klebsiella pneumoniae* Strain

**DOI:** 10.3390/microorganisms14102267

**Published:** 2026-10-05

**Authors:** Sneha Kumkum, Michelle Chioma Kalu, Annie Wong-Beringer

**Affiliations:** 1 Titus Family Department of Clinical Pharmacy, Alfred E. Mann School of Pharmacy and Pharmaceutical Sciences, University of Southern California, Los Angeles, CA 90033, USA; 2 Department of Pharmacy, Huntington Health, Pasadena, CA 91105, USA

**Keywords:** *K. pneumoniae*, plasmid conjugation, antimicrobial resistance, *blaKPC*, horizontal gene transfer, meropenem, gepotidacin, omadacycline

## Abstract

Horizontal transfer of *blaKPC*-bearing plasmids contributes significantly to the dissemination of carbapenem resistance among *Enterobacterales*; however, the influence of mechanistically distinct antibiotics on this process remains incompletely understood. This study investigated the effects of meropenem, gepotidacin, and omadacycline on plasmid-mediated transfer of *blaKPC* from the carbapenem-resistant *K*. *pneumoniae* donor strain CDC120 to the ESBL-producing *K*. *pneumoniae* recipient strain DS073. Conjugation assays were performed under sub-inhibitory antibiotic concentrations, and plasmid transfer was confirmed by PCR and antimicrobial susceptibility testing. Expression of the conjugation-associated genes *traT*, *traS*, *traN***,** and *traG* was quantified by quantitative real-time PCR (qRT-PCR). Meropenem produced the highest conjugation frequency (3.07 × 10^−7^ transconjugants per donor), followed by gepotidacin (4.55 × 10^−8^) and omadacycline (3.38 × 10^−9^). These differences were reflected phenotypically, with meropenem-selected transconjugants demonstrating the greatest increase in meropenem minimum inhibitory concentration (MIC), followed by gepotidacin and omadacycline. Gene expression analysis revealed antibiotic-specific regulation of conjugation-associated genes rather than uniform activation of the conjugation machinery. Among the genes examined, *traS* exhibited the strongest induction across all antibiotic treatments, whereas *traT* was consistently downregulated and *traN* and *traG* displayed treatment-dependent expression patterns. Collectively, these findings demonstrate that antibiotics differentially influence plasmid transfer efficiency and the expression of conjugation-associated genes, thereby affecting the dissemination of *blaKPC*-mediated carbapenem resistance.

## 1. Introduction

Antimicrobial resistance (AMR) poses a significant threat to global public health, compromising the effective treatment of bacterial infections and contributing substantially to patient morbidity, mortality, and healthcare costs worldwide [1,2]. Among multidrug-resistant (MDR) Gram-negative pathogens, carbapenem-resistant *K*. *pneumoniae* (CRKP) has emerged as one of the most clinically significant healthcare-associated pathogens, causing a range of infections, including bacteremia, pneumonia, urinary tract infections, and intra-abdominal infections, that frequently result in poor clinical outcomes [2,3]. The rapid global dissemination of CRKP has been largely driven by plasmid-mediated acquisition of carbapenemase genes, particularly *blaKPC* which encodes the *K. pneumoniae* carbapenemase capable of hydrolyzing nearly all β-lactam antibiotics, including carbapenems [3].

Horizontal gene transfer (HGT) is the principal mechanism responsible for the dissemination of plasmid-mediated antimicrobial resistance among bacterial populations. Among the three major HGT mechanisms, bacterial conjugation is considered the most efficient route for plasmid transfer among members of the *Enterobacterales* family, enabling rapid dissemination of clinically important resistance determinants between genetically unrelated bacteria [4,5]. Conjugative plasmids encode a type IV secretion system that mediates direct DNA transfer through coordinated expression of transfer (*tra*) genes. Proteins encoded by *traG*, *traN*, *traS*, and *traT* genes regulate DNA processing, mating pair stabilization, plasmid transport, and entry exclusion, collectively determining the efficiency of plasmid-mediated gene transfer [5,6,7]. Consequently, increased conjugation efficiency may accelerate dissemination of multidrug resistance among clinical bacterial populations. Increasing evidence suggests that antibiotic exposure itself influences bacterial conjugation in addition to selecting for resistant organisms. Exposure to sub-inhibitory antibiotic concentrations has been shown to activate bacterial stress responses, alter global transcriptional programs, and modulate the expression of conjugation-associated genes, thereby affecting horizontal transfer of resistance plasmids [8].

Recent work from our group demonstrated that recurrent infections caused by extended-spectrum β-lactamase (ESBL)-producing *E*. *coli* and *K*. *pneumoniae* may evolve into distinct carbapenem-resistant phenotypes through acquisition of carbapenemase-encoding plasmids or alternative resistance mechanisms, emphasizing the dynamic in-human evolution of antimicrobial resistance during recurrent infection and the importance of understanding factors that promote dissemination of resistance determinants [9]. While these findings established the clinical significance of resistance evolution, the mechanisms governing subsequent plasmid-mediated dissemination of *blaKPC* between bacterial populations remain poorly understood. In particular, the extent to which different antibiotic classes used for the treatment of infections caused by ESBL-producing Enterobacterales influence conjugative *blaKPC*-plasmid transfer and expression of conjugation-associated genes has not been systematically investigated. Understanding how antimicrobial exposure affects the plasmid-mediated dissemination of *blaKPC* is therefore essential to inform antimicrobial stewardship strategies aimed at limiting the horizontal spread of carbapenem resistance.

Horizontal gene transfer through bacterial conjugation is a major mechanism driving the dissemination of antimicrobial resistance genes, particularly those encoded on conjugative plasmids such as *blaKPC*. The transfer of these plasmids facilitates the rapid spread of carbapenem resistance among clinically important Enterobacterales and contributes substantially to the global burden of multidrug-resistant infections. Recent evidence suggests that exposure to sub-inhibitory concentrations of antibiotics can influence conjugative plasmid transfer, although the magnitude and direction of this effect depend on the antibiotic, bacterial strains, and experimental conditions. For example, Kondratieva et al. demonstrated that sub-inhibitory meropenem exposure altered the transfer efficiency of *blaKPC*- and *blaOXA*-48-bearing plasmids in *K*. *pneumoniae* in a concentration-dependent manner, highlighting the complex relationship between antibiotic exposure and horizontal gene transfer. More broadly, reviews of horizontal gene transfer emphasize that antibiotic-induced stress responses, plasmid biology, and host-specific factors collectively regulate the dissemination of antimicrobial resistance genes [10,11].

The objective of this study was to compare the effects of meropenem, omadacycline, and gepotidacin on plasmid-mediated transfer of *blaKPC* from a CRKP donor strain to an ESBL-producing *K*. *pneumoniae* recipient clinical strain. The agents selected for this study are ESBL-active antibiotics representing mechanistically distinct classes. Meropenem is a broad-spectrum carbapenem that inhibits bacterial cell wall synthesis and remains a guideline-recommended therapy for severe infections caused by carbapenem-resistant *Enterobacterales* (CRE). Omadacycline, an aminomethylcycline antibiotic, inhibits bacterial protein synthesis through binding to the 30S ribosomal subunit and has demonstrated potent in-vitro activity against ESBL-producing *E*. *coli* and *K*. *pneumoniae*, making it a promising oral treatment option for multidrug-resistant Enterobacterales [12]. More recently, gepotidacin, a first-in-class triazaacenaphthylene antibiotic, has emerged as a new alternative oral treatment for uncomplicated urinary tract infections caused by ESBL-producing and multidrug-resistant *E*. *coli* and *K*. *pneumoniae* isolates [13]. This antibiotic targets bacterial DNA gyrase and topoisomerase IV through a binding site distinct from that of fluoroquinolones. This unique dual-target mechanism disrupts bacterial DNA replication while maintaining activity against many resistant Gram-negative pathogens. Because gepotidacin exerts a mechanism of action different from β-lactams and tetracyclines, it provides an opportunity to investigate whether antibiotics with distinct cellular targets differentially influence conjugative plasmid transfer and the dissemination of antimicrobial resistance genes such as *blaKPC* [13]. Despite its novel mechanism of action and promising activity against multidrug-resistant Enterobacterales, little is currently known about the impact of gepotidacin on horizontal gene transfer and conjugative dissemination of antimicrobial resistance plasmids.

We performed conjugation assays, molecular and phenotypic characterization of transconjugants to evaluate (i) antibiotic-dependent differences in conjugation frequency, (ii) changes in the expression of key conjugation-associated genes (*traT*, *traS*, *traG*, and *traN*), and (iii) the acquisition of phenotypic carbapenem resistance following plasmid transfer. We demonstrated that mechanistically distinct antibiotics differentially influence plasmid-mediated dissemination of *blaKPC*, resulting in the acquisition of carbapenem phenotypic resistance with corresponding upregulation of conjugation-associated gene expression in the transconjugants ready for onward dissemination. These findings provide new insights into how antibiotic exposure may influence horizontal gene transfer and have important implications for antimicrobial stewardship strategies aimed at limiting the spread of carbapenem resistance.

## 2. Materials and Methods

The overall design of this study was to investigate the effects of three different antibiotics (meropenem, omadacycline, and gepotidacin) on plasmid-mediated horizontal transfer of *blaKPC* between clinical *K*. *pneumoniae* isolates. Experimental analyses included conjugation assays, PCR confirmation of plasmid acquisition, quantitative real-time PCR (qRT-PCR) analysis of conjugation-associated gene expression, and phenotypic characterization of recovered transconjugants.

### 2.1. Bacterial Strains and Plasmid Characterization

The donor strain (CDC120) was a *blaKPC*-positive carbapenem-resistant *K. pneumoniae* strain obtained from the *Enterobacterales* Carbapenemase Diversity panel from the CDC & FDA Antimicrobial Resistance Isolate Bank (CDC & FDA Antimicrobial Resistance Isolate Bank. Atlanta (GA): CDC.) The recipient strain (DS073) was an ESBL-producing *K. pneumoniae* strain isolated from a patient with recurrent urinary tract infection which later evolved to become *blaKPC*-positive carbapenem resistant upon subsequent isolation. *E. coli* ATCC 25922 was obtained from the American Type Culture Collection and was used as quality control for broth microdilution testing. All strains were stored in 50% glycerol stocks at −80 °C, plated on CHROMagar and incubated at 37 °C, and inoculated in LB Lennox broth (Sigma-Aldrich, St. Louis, MO, USA) and incubated at 37 °C with shaking of 250 rpm. Whole-genome sequencing was performed on the donor–recipient strain pair to characterize plasmid incompatibility groups and assess compatibility between donor and recipient strains for conjugative plasmid transfer. Genome assemblies were examined using Geneious (Geneious Prime 2024.0.4, GraphPad, Boston, MA, USA) to identify plasmid replicon types and the distribution of antimicrobial resistance determinants.

### 2.2. Plasmid Incompatibility Analysis

Plasmid incompatibility analysis was conducted using Geneious Prime (Biomatters Ltd., Auckland, New Zealand) to characterize the plasmids present in the donor strain and transconjugants. Whole-genome sequencing (WGS) assemblies from each isolate were imported into Geneious Prime, where plasmid-associated contigs were identified and annotated. Plasmid sequences were aligned against reference plasmid replicon sequences representing established incompatibility (Inc) groups to determine the incompatibility type of each plasmid. The annotated plasmid sequences were further examined to identify conjugative transfer (*tra*) genes and to confirm the presence of the *blaKPC* carbapenemase gene within the transferred plasmid. Plasmid incompatibility analysis was performed to characterize the genetic background of the conjugative plasmid and verify the conservation of its conjugation machinery following horizontal transfer.

### 2.3. Fluorescent Labeling of Recipient Strain

To facilitate identification of recipient-derived transconjugants following conjugation, the recipient strain DS073 was transformed with a plasmid expressing the DsRedExpress fluorescent protein by electroporation as previously described (13). Electrocompetent cells were prepared by repeated washing with ice-cold 10% glycerol and electroporated with approximately 300 ng of plasmid DNA using a Bio-Rad Micropulser Electroporator (Bio-Rad Laboratories, Hercules, CA, USA) under optimized conditions.

Following electroporation, cells were recovered in Luria–Bertani (LB) broth for 1 h at 37 °C with shaking (250 rpm) before plating on LB agar supplemented with 25 μg/mL zeocin for selection of transformants. Plates were incubated overnight at 37 °C, and fluorescent colonies were confirmed using fluorescence imaging. Stable DsRedExpress-positive transformants were used as recipient strains in all subsequent conjugation assays to enable rapid differentiation of recipient-derived transconjugants from donor cells following antibiotic selection.

### 2.4. Antimicrobial Susceptibility Testing

Minimum inhibitory concentrations (MICs) of meropenem, omadacycline, and gepotidacin were determined for the donor and recipient strains using the broth microdilution method in accordance with Clinical and Laboratory Standards Institute (CLSI) guidelines [14]. Briefly, cation-adjusted Mueller–Hinton broth was inoculated with single colonies and incubated at 37 °C with shaking. Overnight bacterial cultures were adjusted to a 0.5 McFarland standard, diluted in cation-adjusted Mueller–Hinton broth (CAMHB), and inoculated into 96-well microtiter plates containing two-fold serial dilutions of each antibiotic. Plates were incubated at 37 °C for 18–20 h, and MICs were recorded as the lowest antibiotic concentration that completely inhibited visible bacterial growth. *E*. *coli* ATCC 25922 was included as the quality-control strain for susceptibility testing.

MIC values were used to confirm the antimicrobial susceptibility profiles of the donor and recipient strains and to determine sub-inhibitory concentrations (sub-MICs) used in subsequent conjugation assays. Recipient-specific sub-MICs of meropenem, omadacycline, and gepotidacin were subsequently selected for all comparative conjugation experiments. Additionally, MIC values were determined for PCR-confirmed transconjugants later and compared with those of the parental donor and recipient strains to assess phenotypic changes in antimicrobial susceptibility following plasmid transfer.

### 2.5. Conjugation Assays

Conjugation assays were performed to evaluate transfer of the *blaKPC*-harboring plasmid from the carbapenem-resistant donor strain CDC120 to the fluorescently labeled ESBL-producing recipient strain DS073-DsRed. Overnight cultures of donor and recipient strains were grown in Luria–Bertani (LB) broth at 37 °C with shaking, adjusted to an OD_600_ of 0.5, washed with Dulbecco’s phosphate-buffered saline (DPBS), and mixed at a 4:1 donor-to-recipient ratio. A 50 μL aliquot of each mating mixture was spotted onto LB agar containing sub-MICs of meropenem, omadacycline, or gepotidacin and incubated at 37 °C for 18 h. Meropenem and omadacycline experiments were performed at 0.5× of the recipient MIC, whereas gepotidacin experiments were performed using 0.25× and 0.125× of the recipient MIC.

Following incubation, bacterial growth was collected from each mating spot, resuspended in sterile DPBS, serially diluted, and plated onto non-selective LB agar to determine total viable bacterial counts and onto double-selective LB agar supplemented with 25 μg/mL zeocin and 4× the recipient MIC of the corresponding antibiotic to selectively recover recipient-derived transconjugants carrying the transferred *blaKPC* plasmid. Plates were incubated overnight at 37 °C, and fluorescent colonies were confirmed by fluorescence imaging. Representative transconjugants were stored in 50% glycerol at −80 °C for subsequent molecular and phenotypic analyses. Conjugation frequency was calculated by dividing the number of transconjugant colony-forming units (CFU) recovered on double-selective media by the total viable CFU recovered on non-selective LB agar. Each experiment was performed using at least three independent biological replicates.

### 2.6. Molecular Confirmation of blaKPC Plasmid Transfer

Putative transconjugants recovered from double-selective media were screened for acquisition of the *blaKPC* gene by polymerase chain reaction (PCR). Genomic DNA was extracted from overnight cultures using the QuickExtract™ DNA Extraction Solution (Lucigen, Middleton, WI, USA) according to the manufacturer’s instructions. PCR amplification was performed using primers specific for *blaKPC* and *rpoD* (housekeeping gene) listed in Table 1. PCRs were prepared using GoTaq^®^ Green Master Mix (Promega, Madison, WI, USA). PCR products were amplified under standard cycling conditions consisting of an initial denaturation at 95 °C, followed by 30 amplification cycles of denaturation, primer annealing at a temperature of 55 °C, and extension, with a final extension step at 72 °C. Amplified products were separated by electrophoresis on a 1% agarose gel containing GelRed nucleic acid stain and visualized under ultraviolet illumination using a Bio-Rad GelDoc imaging system (Bio-Rad Laboratories, Hercules, CA, USA).

The carbapenem-resistant donor strain CDC120 and the ESBL recipient strain DS073 served as positive and negative controls, respectively. Successful amplification of the expected *blaKPC* and *rpoD* amplicon in transconjugants confirmed acquisition of the carbapenemase-encoding plasmid following conjugation. Primer sequences were designed using Integrated DNA Technologies (IDT, Coralville, IA, USA).

### 2.7. Gene Expression Analysis of Transconjugants (TCs) by Quantitative Real-Time PCR (RT-qPCR)

The expression of conjugation-associated genes was quantified to evaluate gene expression in transconjugants following acquisition of the *blaKPC*-bearing plasmid. Following conjugation, two representative transconjugant colonies recovered from each antibiotic treatment were selected from the selective agar plates and designated TC1 and TC2. Each colony was reconfirmed by growth on LB agar supplemented with 25 μg/mL zeocin and 4× MIC of the corresponding selecting antibiotic prior to gene expression analysis.

Individual colonies were cultured overnight in LB broth at 37 °C with shaking at 250 rpm, followed by subculture and incubation for 6 h under the same conditions before RNA extraction.

Total RNA was extracted using the RNeasy Mini Kit (Qiagen, Hilden, Germany) according to the manufacturer’s instructions. Residual genomic DNA was removed by DNase treatment, and RNA concentration and purity were assessed spectrophotometrically before complementary DNA (cDNA) synthesis.

Complementary DNA was synthesized using the iScript™ Reverse Transcription Supermix (Bio-Rad, Hercules, CA, USA). Quantitative real-time PCR (RT-qPCR) was performed using SsoAdvanced™ Universal SYBR^®^ Green Supermix (Bio-Rad) on a CFX96 Touch Real-Time PCR Detection System (Bio-Rad). Primers targeting *traT*, *traS*, *traG*, *traN*, and *blaKPC* (Table 1 and Table 2) were designed using the Integrated DNA Technologies (IDT) PrimerQuest™ Tool (Integrated DNA Technologies, Coralville, IA, USA) and synthesized by the same manufacturer.

Relative gene expression was normalized to the housekeeping gene *rpoD* and calculated using the 2^−ΔΔCt^ method. For *tra* gene expression analysis, the recipient strain DS073 served as the reference calibrator (fold change = 1). Each RT-qPCR reaction was performed in technical duplicate.

**Table 2:** Primer sequences for *tra genes*.

Gene	Primer Sequence (5′–3′)	Amplicon Size (bp)
*traT*	F: TCTGGCTGGAACCTTCAAATA	127
	R: CTGACGACCTGATATCCCTTTG	
*traS*	F: TAAGGCGAGTTAGTGGTTGTG	142
	R: CCTGACATAGGTAACCCTGTTG	
*traN*	F: TCTCAGGATGCACCGTTTATT	140
	R: AGATCACGTTCGCAGGTATG	
*traG*	F: TATCAACAGGGCCGTGAAAG	129
	R: ACCAGCATCTTGCGTCATAG	

### 2.8. Statistical Analysis

Statistical analyses (one-way ANOVA followed by Tukey’s multiple-comparison test) were performed only for conjugation frequency data. Gene expression analyses were descriptive because RT-qPCR measurements were obtained from technical duplicates rather than independent biological replicates. All statistical analyses including one-way ANOVA and Tukey’s multiple comparison tests were performed using GraphPad Prism 11.0.2. (GraphPad Software, San Diego, CA, USA). Differences between experimental groups were considered statistically significant at *p* ≤ 0.05.

## 3. Results

### 3.1. Selection of a Compatible Donor–Recipient Pair for Conjugation Studies

Whole-genome sequencing and plasmid incompatibility analysis identified the carbapenem-resistant *blaKPC* plasmid bearing donor strain CDC120, carrying an IncA/C plasmid, and the ESBL-producing clinical isolate DS073, carrying an IncFIA plasmid, as a compatible donor–recipient pair for all subsequent conjugation experiments. Additionally, PCR analysis confirmed that the recipient strain DS073 did not harbor the *blaKPC* gene prior to conjugation, ensuring that detection of *blaKPC* in transconjugants represented a direct result from successful plasmid transfer.

### 3.2. Antimicrobial Susceptibility Profiles of Donor and Recipient Strains

The antimicrobial susceptibility profiles of the donor strain (CDC120) and recipient strain (DS073) were determined prior to conjugation experiments (Table 3). The donor strain exhibited resistance to meropenem, with an MIC of 16 µg/mL, consistent with carriage of the *blaKPC*-containing plasmid. In contrast, the recipient strain (DS073) was susceptible to meropenem, with an MIC of 0.125 µg/mL, confirming its suitability as a recipient for evaluating plasmid-mediated acquisition of carbapenem resistance. Susceptibility testing further demonstrated distinct antimicrobial susceptibility profiles for omadacycline and gepotidacin. The quality control strain, *E*. *coli* ATCC 25922, exhibited MIC values within the expected reference ranges for the study antibiotics.

### 3.3. Antibiotic-Specific Effects on Conjugation Frequency

**Figure 1:**
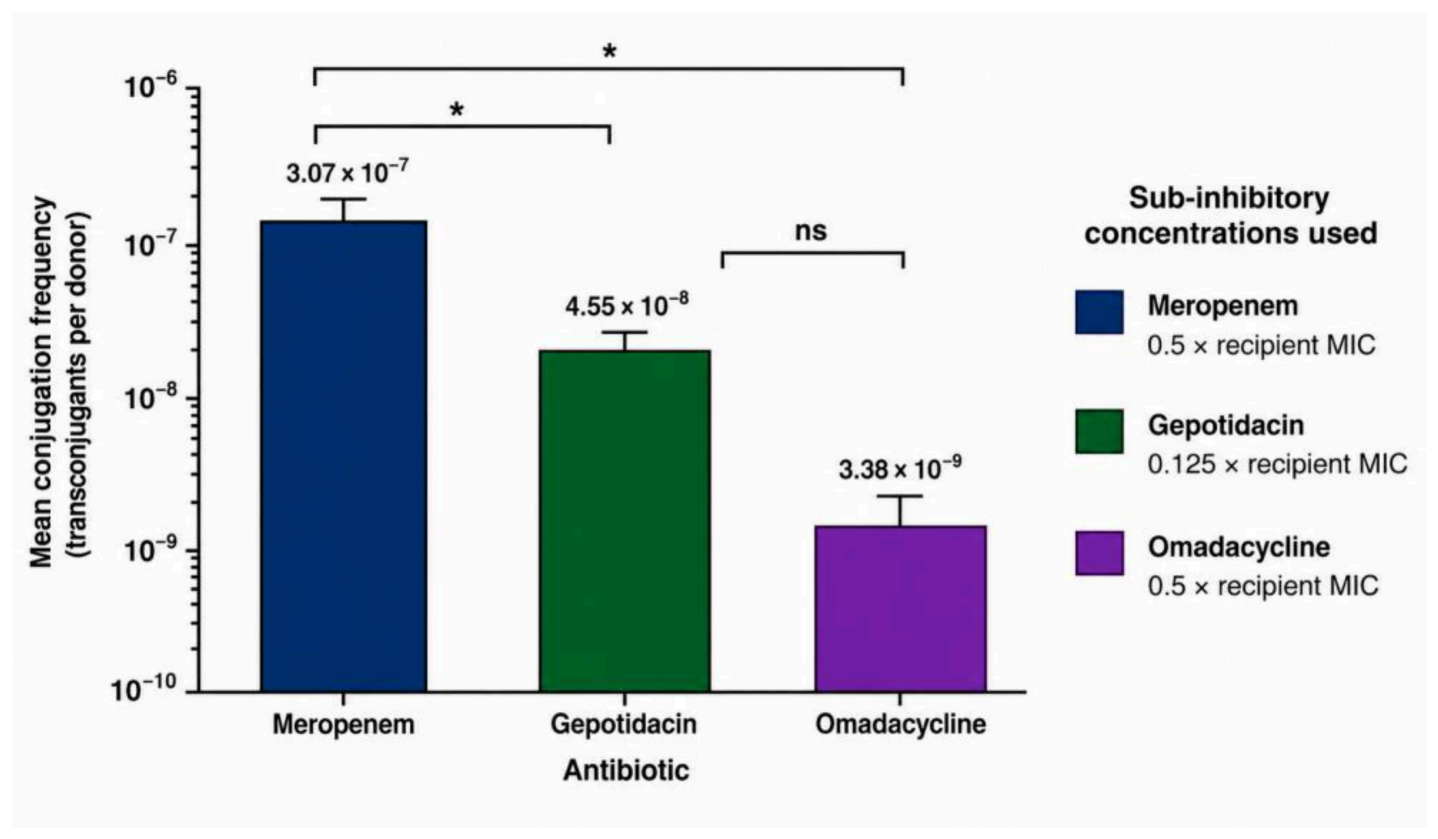
Comparative analysis of conjugation frequency under different antibiotic exposure. Figure 1 presents the effect of sub-inhibitory antibiotic exposure on conjugative transfer of the *blaKPC*-bearing plasmid from CDC120 to DS073. Conjugation assays were performed using meropenem and omadacycline at 0.5× the recipient MIC, whereas gepotidacin was evaluated at 0.125× the recipient MIC. Bars represent the mean conjugation frequency measured at the indicated sub-inhibitory antibiotic concentration and are expressed as mean ± SD of biological replicates. Statistical significance was determined by one-way ANOVA followed by Tukey’s multiple-comparison test. *p* < 0.05; ns, not significant. Bars represent the mean conjugation frequency measured at the indicated sub-inhibitory antibiotic concentration and are expressed as mean ± SD of biological replicates. Brackets indicate pairwise comparisons between treatment groups. * Indicates a statistically significant difference (*p* < 0.05), whereas ns (not significant) indicates that no statistically significant difference was observed between the compared groups (*p* ≥ 0.05).

Sub-inhibitory exposure to meropenem, gepotidacin, and omadacycline differentially affected *blaKPC* plasmid conjugation frequencies. PCR amplification confirmed the presence of *blaKPC* in transconjugants recovered following exposure to all three antibiotics, confirming successful plasmid transfer under each experimental condition. Meropenem and omadacycline conjugation assays were performed at 0.5× the recipient MIC, whereas gepotidacin assays were performed at 0.125× the recipient MIC. The conjugation frequencies presented correspond to the sub-inhibitory concentration that yielded the highest transfer frequency for each antibiotic. PCR amplification confirmed the presence of blaKPC in transconjugants recovered following exposure to all three antibiotics, confirming successful plasmid transfer under each experimental condition. Meropenem produced the highest conjugation frequency (3.07 × 10^−7^ transconjugants per donor), followed by gepotidacin (4.55 × 10^−8^) and omadacycline (3.38 × 10^−9^) (Figure 1). Statistical analysis using one-way ANOVA followed by Tukey’s multiple-comparison test demonstrated that meropenem significantly increased conjugation frequency compared with both gepotidacin and omadacycline (*p* < 0.05), whereas the difference between gepotidacin and omadacycline was not statistically significant (*p* > 0.05). These findings demonstrate that antibiotic class differentially influences blaKPC plasmid transfer under sub-inhibitory conditions.

### 3.4. blaKPC Expression and Phenotypic Confirmation of Antimicrobial Resistance in Transconjugants

**Figure 2:**
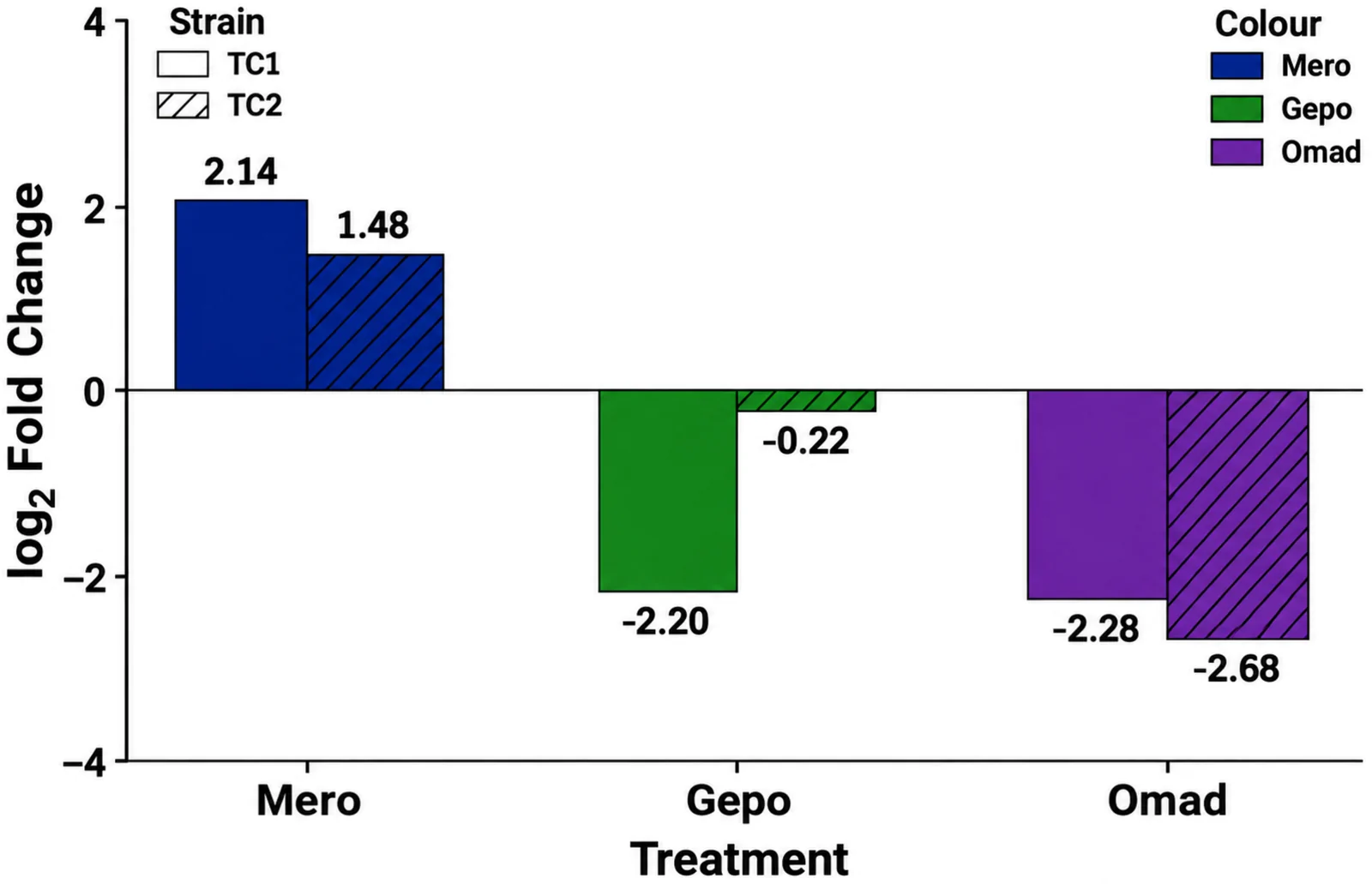
Relative *blaKPC* expression in meropenem-, gepotidacin-, and omadacycline-selected transconjugants compared to donor strain.

Relative *blaKPC* expression was evaluated in transconjugants recovered following exposure to all three antibiotics and showed that transferred plasmids remained transcriptionally active following conjugation regardless of antibiotic exposure (Figure 2). However, differences in *blaKPC* expression were observed among treatment groups which followed similar pattern as conjugation frequencies. Following meropenem selection, *blaKPC* expression was increased in both transconjugants showing a log_2_ fold change of +2.14 to +1.48 which corresponded to approximately 4.4-fold and 2.8-fold increases in expression, respectively, relative to the donor strain. In contrast, gepotidacin selection was associated with slightly reduced *blaKPC* expression with the transconjugants showing a log_2_ fold change of −2.20 to −0.22, corresponding to approximately 0.22-fold to 0.86-fold reduced expression. Omadacycline-mediated transconjugants expressed the lowest level of *blaKPC* in both transconjugants with log_2_ fold changes of −2.28 and −2.68, corresponding to approximately 0.21-fold and 0.16-fold expression, respectively. Overall, when compared to donor strain, meropenem-selected transconjugants exhibited upregulated *blaKPC* expression while both gepotidacin- and omadacycline-selected transconjugants showed downregulated expression with omadacycline showing stronger suppression.

Relative *blaKPC* expression was quantified in two transconjugants, TC1 and TC2, following selection under meropenem, gepotidacin, and omadacycline conditions. Gene expression was normalized to the housekeeping gene *rpoD* and calculated using the $2^{-\Delta \Delta Ct}$ method. Values are shown as log_2_ fold change relative to the donor strain, where 0 corresponds to a fold change of 1. Positive values indicate increased expression, whereas negative values indicate reduced expression. Solid bars represent TC1 and diagonally lined bars represent TC2. Meropenem, gepotidacin, and omadacycline are shown in blue, green, and purple, respectively. No inferential statistical testing was performed because the RT-qPCR measurements were based on technical duplicates rather than independent biological replicates.

In addition, MIC testing was performed to determine whether acquisition of the *blaKPC*-carrying plasmid resulted in phenotypic resistance to meropenem in the transconjugants (Table 4). Transconjugants recovered following meropenem exposure showed an increase in meropenem MIC of the recipient strain by 128-fold from 0.125 to 16 µg/mL, reaching the same level of resistance as the donor strain. These findings confirmed successful acquisition and functional expression of carbapenem resistance following plasmid transfer. In comparison, *blaKPC*-carrying transconjugants recovered following exposure to gepotidacin increased meropenem MIC to 8 µg/mL while omadacycline exposure increased meropenem MIC to 4 µg/mL. Notably, gepotidacin MICs of the transconjugants remained comparable to or slightly greater (2–4 µg/mL) than those of the donor and recipient strains (MIC of 2 µg/mL), indicating that *blaKPC* plasmid acquisition did not substantially alter susceptibility to gepotidacin. Interestingly, transconjugants showed greater susceptibility to omadacycline following *blaKPC* plasmid uptake upon exposure across all three antibiotics, while gaining resistance to meropenem. A reduction in omadacycline MIC by 2–8 fold from 16 µg/mL of the recipient pre-transfer to as low as 2 µg/mL in the transconjugants was observed, showing an inverse relationship between meropenem and omadacycline resistance following plasmid uptake.

Although *blaKPC* expression was reduced in the gepotidacin- and omadacycline-selected transconjugants relative to the donor strain (CDC120), these isolates nevertheless exhibited elevated meropenem MICs compared with the recipient strain DS073. This indicates that reduced *blaKPC* transcript abundance did not eliminate the carbapenem-resistant phenotype following plasmid acquisition.

Together, these findings demonstrate that conjugative transfer resulted in both the *blaKPC* acquisition and phenotypic expression of meropenem resistance. The marked increase in meropenem MICs in the transconjugants confirms successful transfer of functional carbapenem resistance mediated by the *blaKPC*-encoding plasmid to varying degree specific to the antibiotic agent exposed. Meropenem was the strongest inducer of *blaKPC*-plasmid transfer resulting in the highest phenotypic expression of meropenem resistance while omadacycline showed the lowest induction potential and phenotypic expression accompanying by collateral sensitivity to omadacycline as meropenem resistance is expressed.

### 3.5. Antibiotic-Mediated Effects on tra Gene Expression

**Figure 3:**
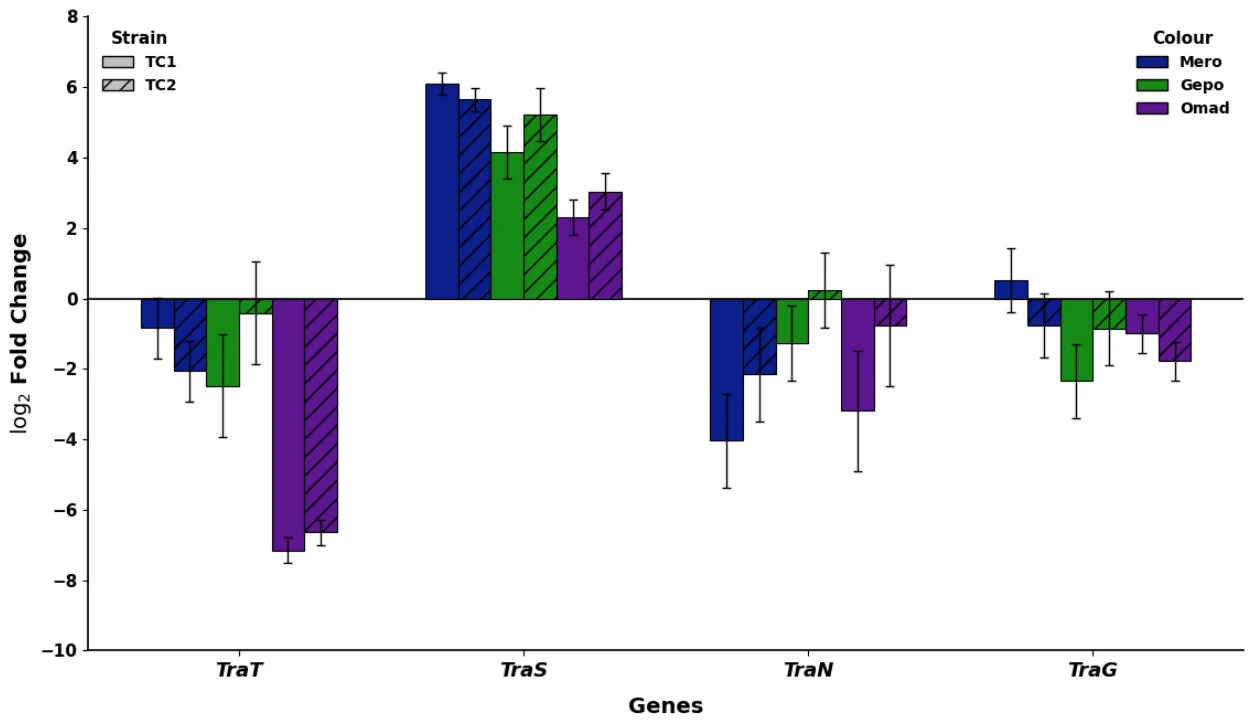
Expression of conjugation-associated genes in transconjugants following antibiotic exposure.

We explored whether antibiotic exposure alters the activation level of the conjugation machinery in the newly formed plasmid-bearing cells enabling onward dissemination. The relative expression of *traT*, *traS*, *traG*, and *traN* in two representative transconjugants recovered from exposure to each antibiotic was quantified by RT-qPCR and expressed as log_2_fold change relative to the untreated recipient control (DS073 RFP) (Figure 3).

Among the genes examined, antibiotic exposure predominantly activated *traS*, while *traT* and *traN* were repressed. *traG* remained relatively stable showing only modest changes. The response was antibiotic-dependent. Both transconjugants exhibited similar transcriptional responses to each antibiotic, although minor differences in the magnitude of expression was observed.

*traS* was consistently the most highly induced following exposure to all three antibiotics. Meropenem was associated, with an increase of 48–64 fold, followed by gepotidacin (17–36 fold). Omadacycline resulted in a comparatively lower induction (5–8 fold) in both transconjugants. In contrast, *traT* was downregulated under all antibiotic treatments. Omadacycline caused the greatest repression, reducing expression by approximately 100–150-fold, whereas meropenem and gepotidacin produced more moderate decreases of approximately 2–6-fold. Similarly, *traN* expression was reduced following antibiotic exposure. Meropenem produced the strongest repression (approximately 16-fold), while gepotidacin and omadacycline caused moderate decreases ranging from approximately 2–8-fold. In TC2, gepotidacin-treated cells exhibited expression levels close to the untreated control. Compared with the other genes, *traG* displayed relatively small changes in expression. Meropenem caused little or no change, whereas gepotidacin and omadacycline resulted in modest reductions of approximately 2–4-fold. Overall, the transcriptional responses of TC1 and TC2 were highly comparable across all antibiotics.

Relative expression of conjugation-associated genes in antibiotic-selected transconjugants. Expression of *traT*, *traS*, *traN*, and *traG* was measured in two transconjugants, TC1 and TC2, selected under meropenem, gepotidacin, and omadacycline conditions. Gene expression is presented as log_2_ fold change relative to the DS073 RFP recipient strain. Positive values indicate increased expression, while negative values indicate reduced expression relative to DS073 RFP. Solid bars represent TC1 and diagonal lined bars represent TC2. Error bars represent variability between technical duplicates.

Overall, the data demonstrate that antibiotic exposure produced gene-specific effects rather than uniform regulation of all conjugation-associated genes in the transconjugants. *traS* was most strongly upregulated under meropenem followed by gepotidacin and omadacycline, in the newly formed plasmid-bearing cells closely mirrored the conjugation frequency results shown in Figure 1. On the other hand, *traT*, *traN*, and *traG* showed variable patterns of downregulation depending on the antibiotic. Similar expression patterns were observed in both representative transconjugants.

## 4. Discussion

The rapid global dissemination of carbapenem-resistant Enterobacterales (CRE) is largely driven by the horizontal transfer of plasmid-mediated resistance determinants such as *blaKPC*. Although bacterial conjugation is recognized as a major mechanism underlying the spread of antimicrobial resistance, the influence of different antibiotic classes on plasmid transfer remains incompletely understood. In the present study, we investigated the effects of sub-inhibitory exposure to meropenem, gepotidacin, and omadacycline on the conjugative transfer of a *blaKPC*-carrying plasmid from a carbapenem-resistant *K*. *pneumoniae* donor strain to an ESBL-producing *K*. *pneumoniae* clinical recipient strain. Our findings demonstrated that antibiotic exposure differentially influenced conjugation frequency, expression of conjugation-associated genes, and acquisition of phenotypic carbapenem resistance.

Among the antibiotics evaluated, meropenem exposure resulted in the highest conjugation frequency, followed by gepotidacin, whereas omadacycline produced the lowest frequency of plasmid transfer. These findings indicate that different antibiotic classes can differentially influence the horizontal dissemination of resistance plasmids. Similar observations with meropenem exposure were reported by Kondratieva et al., who investigated the effect of meropenem on conjugative plasmid transfer using clinical *K*. *pneumoniae* donor and recipient strains carrying either IncFII *blaKPC-2* or IncL *blaOXA-48* plasmids [8]. Their study demonstrated that the effect of meropenem was dependent on both antibiotic concentration and the donor–recipient pair, with exposure to 0.5× MIC generally resulting in higher conjugation frequencies than those observed in untreated controls. The authors proposed that antibiotic-induced stress responses, including activation of the bacterial SOS response, may contribute to enhanced plasmid dissemination under sub-inhibitory antibiotic exposure. Although these mechanisms were not directly investigated in the present study, our findings are consistent with those of Kondratieva et al., who similarly observed enhanced plasmid transfer following sub-inhibitory meropenem exposure. In addition, our comparison of meropenem, gepotidacin, and omadacycline extends these observations by demonstrating that the magnitude of conjugation transfer differs among the antibiotic classes evaluated [8].

Considering that these antibiotics have distinct mechanisms of action compared with β-lactams, the cellular responses induced by each antimicrobial class may differentially influence conjugative transfer. Although the precise molecular mechanisms were not investigated in the present study, these observations suggest that antibiotic-specific modulation of plasmid transfer extends beyond carbapenem exposure and may vary according to the physiological responses elicited by individual antibiotics. One mechanism that may contribute to the increased plasmid transfer observed following meropenem exposure is activation of the bacterial SOS response. Previous studies have shown that antibiotic-induced DNA damage can stimulate expression of genes involved in conjugative transfer through activation of stress-response pathways. Although SOS response regulators were not evaluated in the present study, the observed increase in conjugation frequency and conjugation-associated gene expression following meropenem exposure likely involved this proposed mechanism. Additional studies investigating regulators such as *recA*, *lexA*, and other stress-response pathways to determine their contribution to antibiotic-specific plasmid dissemination would be of interest for future studies.

The *blaKPC* expression pattern in the transconjugants broadly paralleled the conjugation-frequency results. Notably, meropenem exposure resulted the highest *blaKPC* expression in the transconjugants which paralleled the highest conjugation frequency observed among the three antibiotics. The parallel patterns of conjugation frequency and transconjugant *blaKPC* expression suggest that antibiotic conditions favoring plasmid transfer may also be associated with greater expression of the acquired resistance determinant following plasmid establishment. While conjugation frequency reflects successful transfer of the plasmid between donor and recipient cells, expression of plasmid-encoded resistance genes may be influenced by additional regulatory processes within the transconjugant as in the case of gepotidacin and omadacycline-exposure conditions which showed suppressed *blaKPC* expression despite successful plasmid uptake albeit at lower conjugation frequencies. Factors such as plasmid copy number, promoter activity, host–cell regulatory networks, antibiotic-specific stress responses, and plasmid–host interactions may influence the magnitude of *blaKPC* expression after transfer. Further investigation is required to determine the mechanisms responsible for differential *blaKPC* expression following exposure to different antibiotic classes.

Interestingly, despite reduced *blaKPC* expression in the gepotidacin- and omadacycline-selected transconjugants relative to the donor strain, both groups retained elevated meropenem MICs. This observation suggests that maximal *blaKPC* transcription may not be required to confer phenotypic carbapenem resistance following plasmid acquisition. Even at lower transcript levels, sufficient KPC carbapenemase may be produced to hydrolyze meropenem and increase the resistance phenotype. Moreover, antimicrobial susceptibility is influenced by multiple factors beyond transcript abundance, including plasmid copy number, translational efficiency, enzyme activity, and host-specific regulatory mechanisms. Therefore, the relationship between *blaKPC* expression and meropenem MIC is unlikely to be strictly linear, and reduced gene expression does not necessarily correspond to loss of phenotypic resistance.

Phenotypic expression of carbapenem resistance further demonstrated successful acquisition of functional expression of *blaKPC* following conjugation. All confirmed transconjugants exhibited increase in meropenem MICs ranging from 32-fold to 128-fold compared to the recipient strain, dependent on the specific antibiotic to which it was exposed. In contrast, susceptibility of the transconjugants to gepotidacin remained largely unchanged following plasmid acquisition, supporting the absence of KPC-mediated resistance to this antimicrobial agent. Of interest, transconjugants recovered from omadacycline exposure appeared to gain collateral susceptibility to omadacycline in an inverse relationship as they gained resistance to meropenem. It is possible that plasmid uptake and expression of *blaKPC* resistance imposes a fitness cost to the transconjugants or reduced efflux of omadacycline leading to increased susceptibility.

Conjugation is a multistep process involving donor–recipient contact, mating-pair stabilization, plasmid DNA processing, transfer through the conjugative apparatus, and successful establishment of the plasmid in the recipient. The conjugation frequency observed under each antibiotic condition is therefore unlikely to be determined by the expression of any single *tra* gene. Instead, the higher conjugation frequency under meropenem exposure may reflect the combined activity of multiple plasmid-transfer components including other key transfer regulators and additional cellular processes that were not measured in the present study. Once a functional plasmid is established post-transfer, we were interested in whether antibiotic exposure also affected the conjugation machinery in the transconjugants for onward dissemination.

While meropenem exposure created conditions that were most favorable for transfer of the *blaKPC*-carrying plasmid from CDC120 to an ESBL-producing *K*. *pneumoniae* clinical strain, the expression profiles of the conjugation-associated genes in the transconjugants appeared to be more complex than the conjugation-frequency pattern. Among the genes examined, *traS* most closely followed the antibiotic-dependent trend in conjugation frequency. Expression of *traS* was highest following meropenem exposure, intermediate following gepotidacin exposure, and lowest following omadacycline exposure which corresponded to conjugation frequency. This parallel pattern suggests that *traS* may be associated with the antibiotic-dependent differences in plasmid-transfer efficiency. Since the function of *traS* protein is to mediate surface exclusion to avoid redundant plasmid transfer, it is possible that the upregulation of *traS* is an acute response of the newly formed plasmid-bearing cells following plasmid uptake. In contrast, expression of *traT*, *traN*, and *traG* was predominantly downregulated. The decrease in *traT*, particularly under omadacycline, may reduce the ability of the transconjugants to form effective contact with new recipient cells and transfer the plasmid again. However, this must be confirmed by using the transconjugants as donor strains in a second conjugation experiment. Overall, these findings demonstrate that the measured *tra* genes were not regulated as a single coordinated transcriptional group under the experimental conditions.

Overall, meropenem exposure was associated with the highest conjugation frequency and the highest *blaKPC* expression in the resulting transconjugants. Gepotidacin produced an intermediate response, whereas omadacycline generally produced the lowest conjugation frequency and *blaKPC* expression. In contrast, the individual *Tra* genes displayed gene-specific expression patterns, with only *traS* clearly following the same antibiotic-dependent trend as conjugation frequency. These findings demonstrate that plasmid transfer, conjugation-associated gene expression, and resistance-gene expression are related but represent distinct biological processes that should be interpreted separately.

The present study has several limitations. First, conjugation experiments were performed using a single donor–recipient pair consisting of a carbapenem-resistant *K*. *pneumoniae* donor (CDC120) and an ESBL-producing *K*. *pneumoniae* recipient (DS073). While the findings may not be generalizable to genetically diverse clinical isolates, the use of a clinical strain as recipient increases the translational relevance of our findings on antibiotic-specific differences in plasmid transfer. Future studies should include a larger number of donor–recipient combinations to determine whether the observed effects are conserved across different strain backgrounds and plasmid types. Second, the present study focused exclusively on *K*. *pneumoniae*. Expanding these investigations to other clinically important members of the *Enterobacterales* family, including *E*. *coli*, *Enterobacter* spp., and *Citrobacter* spp., would provide a broader understanding of antibiotic-specific plasmid dissemination. It is notable that the RT-qPCR measurements were based on technical duplicates rather than independent biological replicates. The expression error bars therefore represent technical variability and cannot be used to estimate biological variation. Inferential statistical comparisons were consequently not performed for the gene-expression data, and the observed expression differences should be interpreted descriptively rather than as statistically significant. Additional independent biological replicates, a larger number of transconjugants, and additional donor and recipient strain backgrounds will be required to determine whether the similar expression patterns are observed. Finally, although differential expression of a selected panel of conjugation-associated genes and differences in conjugation frequency were observed following antibiotic exposure, the molecular mechanisms underlying these responses were not directly investigated. As activation of the SOS response has been implicated in antibiotic-induced horizontal gene transfer, future studies examining these regulatory pathways will provide further mechanistic insight into the molecular mechanisms underlying antibiotic-dependent modulation of plasmid transfer.

## 5. Limitations and Future Directions

The present study has several limitations that should be considered when interpreting the findings. First, conjugation experiments were performed using a single donor–recipient pair consisting of a carbapenem-resistant *K*. *pneumoniae* donor (CDC120) and an ESBL-producing *K*. *pneumoniae* recipient (DS073). Although the use of clinically relevant isolates increases the translational relevance of the study, the findings may not be generalizable across genetically diverse clinical strains, plasmid incompatibility groups, or bacterial species. Future studies should therefore evaluate multiple donor–recipient combinations representing different plasmid backbones and clinically important members of the Enterobacterales, including *Escherichia coli*, *Enterobacter* spp., and *Citrobacter* spp., to determine whether the antibiotic-specific effects observed here are broadly conserved.

Second, the RT-qPCR analysis was performed using two representative transconjugants recovered from each antibiotic condition, with each reaction performed in technical duplicate. Consequently, the gene-expression data are descriptive and do not represent biological variability across independent conjugation events. Although similar expression trends were observed between TC1 and TC2, future investigations should include a larger number of independently derived transconjugants and independent biological replicates to improve statistical power and strengthen confidence in the observed transcriptional responses.

Third, this study focused on the expression of a selected panel of conjugation-associated genes (*traT*, *traS*, *traG*, and *traN*). However, plasmid transfer is regulated by a complex network of plasmid-encoded and host-encoded factors. Additional genes involved in DNA processing, pilus assembly, plasmid maintenance, stress adaptation, and global transcriptional regulation were not examined. Future transcriptomic approaches, including RNA sequencing, could provide a more comprehensive understanding of antibiotic-dependent changes in the conjugation machinery following plasmid transfer.

Fourth, although antibiotic-specific differences in conjugation frequency were observed, the molecular mechanisms responsible for these differences remain unclear. Previous studies have implicated activation of the SOS response, oxidative stress pathways, alterations in membrane permeability, and changes in plasmid copy number as potential contributors to enhanced horizontal gene transfer under antibiotic exposure. These mechanisms were not directly investigated in the present study. Future work should therefore evaluate regulators such as RecA, LexA, reactive oxygen species (ROS) production, plasmid copy number, and other stress-response pathways to establish the mechanistic basis underlying the observed differences in plasmid dissemination.

Finally, while PCR, antimicrobial susceptibility testing, and gene-expression analysis confirmed successful acquisition and functional expression of the *blaKPC*-bearing plasmid, whole-genome sequencing was not performed on the recovered transconjugants following conjugation to verify complete plasmid architecture or exclude potential structural rearrangements during transfer. Long-read sequencing of transconjugants in future studies would enable confirmation of intact plasmid transmission, identify possible recombination events, and further characterize the genetic stability of transferred resistance plasmids.

Overall, despite these limitations, this study provides evidence that mechanistically distinct antibiotics differentially influence conjugative transfer of a clinically relevant *blaKPC*-bearing plasmid and identifies antibiotic-specific differences in both plasmid dissemination and conjugation-associated gene expression. These findings establish a foundation for future mechanistic investigations and may contribute to the development of antimicrobial stewardship strategies aimed at minimizing plasmid-mediated dissemination of carbapenem resistance.

## 6. Conclusions

This study demonstrates that exposure to mechanistically distinct antibiotic classes active against ESBL-producing Enterobacterales differentially mediates the horizontal uptake of a *blaKPC*-carrying plasmid accompanied by successful acquisition of phenotypic carbapenem resistance in a clinical ESBL-producing *K*. *pneumoniae* recipient strain, with meropenem showing the strongest and omadacycline the least effects. Importantly, differences in the antibiotic-mediated conjugation frequency paralleled the level of *blaKPC* gene and phenotypic resistance. Overall, these findings improve our understanding of how antibiotic exposure influences horizontal dissemination of carbapenem resistance and offer insights that could aid in the development of antimicrobial stewardship strategies aimed at limiting plasmid-mediated dissemination of clinically important resistance determinants.

## Figures and Tables

**Table 1:** Primer sequences for *blaKPC* and *rpoD*.

Gene	Primer Sequence (5′–3′)	Amplicon Size (bp)
*blaKPC*	F: CGTGACGGAAAGCTTACAAA	78
	R: AGCCAATCAACAAACTGCTG	
*rpoD*	F: TCCGGTGCATATGATTGAGA	105
	R: ATACGCTCAGCCAGCTCTTC	

**Table 3:** Minimum inhibitory concentrations (MICs, µg/mL) of meropenem, omadacycline, and gepotidacin against the donor, recipient, and quality control strains used in this study.

Strain	MIC (µg/mL)			Interpretive Criteria
	Meropenem	Omadacycline	Gepotidacin	
CDC120 (Donor)	16	2	2	CLSI: Meropenem ≤1 µg/mL; FDA: Omadacycline ≤4 µg/mL; FDA: Gepotidacin ≤16 µg/mL
DS073 (Recipient)	0.125	16	2	Same as above
*Escherichia coli* ATCC 25922 (Quality Control)	0.06	1	0.05	Quality control strain

**Table 4:** Antibiotic susceptibility profiles on transconjugants (MIC, µg/mL).

Antimicrobial Agent	Donor	Recipient	Transconjugants (TC) Recovered from Antibiotic Exposure					
			Meropenem		Gepotidacin		Omadacycline	
	CDC120	DS073	TC1	TC2	TC1	TC2	TC1	TC2
Meropenem	>16	0.125	>16	16	8	8	4	4
Gepotidacin	2	2	2	2	2	4	2	4
Omadacycline	2	16	2	2	4	2	8	8

## Data Availability

The original contributions presented in this study are included in the article. Further inquiries can be directed to the corresponding author.

## Abbreviations

The following abbreviations are used in this manuscript:

ESBL	Extended-spectrum beta-lactamase
CRKP	Carbapenem-resistant Klebsiella pneumoniae

## References

[1] Halawa E.M., Fadel M., Al-Rabia M.W., Behairy A., Nouh N.A., Abdo M., Fericean L., Atwa A.M., El-Nablaway M., Abdeen A. (2024). Antibiotic action and resistance: Updated review of mechanisms, spread, influencing factors, and alternative approaches for combating resistance. Front. Pharmacol..

[2] Ahmed S.K., Hussein S., Qurbani K., Ibrahim R.H., Fareeq A., Mahmood K.A., Mohamed M.G. (2024). Antimicrobial resistance: Impacts, challenges, and future prospects. J. Med. Surg. Public Health.

[3] Sawa T., Kooguchi K., Moriyama K. (2020). Molecular diversity of extended-spectrum β-lactamases and carbapenemases, and antimicrobial resistance. J. Intensive Care.

[4] San Millan A. (2018). Evolution of plasmid-mediated antibiotic resistance in the clinical context. Trends Microbiol..

[5] Virolle C., Goldlust K., Djermoun S., Bigot S., Lesterlin C. (2020). Plasmid transfer by conjugation in Gram-negative bacteria: From the cellular to the community level. Genes.

[6] Fang L., Chen R., Li C., Sun J., Liu R., Shen Y., Guo X. (2024). The association between the genetic structures of commonly incompatible plasmids in Gram-negative bacteria, their distribution and the resistance genes. Front. Cell. Infect. Microbiol..

[7] Bragagnolo N., Rodriguez C., Samari-Kermani N., Fours A., Korouzhdehi M., Lysenko R., Audette G.F. (2020). Protein dynamics in F-like bacterial conjugation. Biomedicines.

[8] Kondratieva D.A., Savelieva J.R., Golikova M.V. (2024). Effect of meropenem on conjugative plasmid transfer in *Klebsiella pneumoniae*. Int. J. Mol. Sci..

[9] Kruse D., Ny P., Nieberg P., Lou M., Wong-Beringer A. (2026). Recurrent infections caused by ESBL-producing Enterobacterales with or without subsequent development of distinct carbapenem-resistant phenotypes: Differential driving factors and impact on outcome. JAC Antimicrob. Resist..

[10] Michaelis C., Grohmann E. (2023). Horizontal Gene Transfer of Antibiotic Resistance Genes in Biofilms. Antibiotics.

[11] Wiegand I., Hilpert K., Hancock R.E.W. (2008). Agar and broth dilution methods to determine the minimum inhibitory concentration (MIC) of antimicrobial substances. Nat. Protoc..

[12] Stone T.J., Kilic A., Williamson J.C., Palavecino E.L. (2023). In vitro activity of omadacycline and comparator antibiotics against extended-spectrum beta-lactamase-producing *Escherichia coli* and *Klebsiella pneumoniae* urinary isolates. Antibiotics.

[13] Mendes R.E., Scullin A., Kimbrough H., Hubler C., Kapoor R., Torumkuney D., Ryan Arends S.J. (2025). P-1059. In vitro Activity of Gepotidacin against *Klebsiella pneumoniae*, Including Molecularly Characterized ESBL and Carbapenemase Positive Subsets Causing Urinary Tract Infections in the United States (2019–2022). Open Forum Infect. Dis..

[14] Sambrook J., Russell D.W. (2006). Transformation of *E. coli* by electroporation. Cold Spring Harb. Protoc..

